# Anticarcinogenic Effects of Isothiocyanates on Hepatocellular Carcinoma

**DOI:** 10.3390/ijms232213834

**Published:** 2022-11-10

**Authors:** Yuting Zhang, Huiting Huang, Libo Jin, Sue Lin

**Affiliations:** 1 Institute of Life Sciences, College of Life and Environmental Science, Wenzhou University, Wenzhou 325035, China; 2 Biomedicine Collaborative Innovation Center of Zhejiang Province, Wenzhou University, Wenzhou 325035, China

**Keywords:** hepatocellular carcinoma, isothiocyanates, chemoprevention, anticarcinogenic activity, chemosensitization, combination therapies

## Abstract

Hepatocellular carcinoma (HCC) is the most common type of primary liver cancer, accounting for about 90% of cases. Sorafenib, lenvatinib, and the combination of atezolizumab and bevacizumab are considered first-line treatments for advanced HCC. However, clinical application of these drugs has also caused some adverse reactions such as hypertension, elevated aspartate aminotransferases, and proteinuria. At present, natural products and their derivatives have drawn more and more attention due to less side effects as cancer treatments. Isothiocyanates (ITCs) are one type of hydrolysis products from glucosinolates (GLSs), secondary plant metabolites found exclusively in cruciferous vegetables. Accumulating evidence from encouraging in vitro and in vivo animal models has demonstrated that ITCs have multiple biological activities, especially their potentially health-promoting activities (antibacterial, antioxidant, and anticarcinogenic effects). In this review, we aim to comprehensively summarize the chemopreventive, anticancer, and chemosensitizative effects of ITCs on HCC, and explain the underlying molecular mechanisms.

## 1. Introduction

Primary liver cancer is the sixth most common cancer and the third leading cause of cancer-related death worldwide in 2020, with steady growth for nearly two decades [1,2]. The incidence of liver cancer is higher in transitioned countries than that of transitioning countries, and the incidence rate and mortality rate for men are both higher than that for women in most areas [2]. Liver tumors include hepatocellular carcinoma (HCC), intrahepatic cholangiocarcinoma, and other seldom tumors, among which HCC is the most common pathological type accounting for about 90% of cases [3]. Due to the relatively insidious onset and often late diagnosis, most patients with liver cancer are not suitable for surgical resection. For these patients, promising treatment options, as systemic chemotherapy and targeted drug therapy, are available. Currently, the combination of atezolizumab and bevacizumab (an anti-VEGF antibody) has become the standard of care as first-line therapy for advanced HCC, except for patients with contraindications to vascular endothelial growth factor (VEGF) inhibitors and immunotherapy [4,5]. Nevertheless, sorafenib (a small-molecule multikinase inhibitor) and lenvatinib (a multikinase inhibitor) are considered the first-line treatments for advanced-stage HCC patients [6,7]. However, treated with these therapies, patients will have adverse reactions, such as hypertension, elevated aspartate aminotransferase, and proteinuria [8].

At present, more and more studies focus on biologically active natural compounds, especially those extracted from plants with the advantages of low toxicity and less adverse reactions [9,10,11]. In the past two decades, about one-third of FDA-approved drugs have derived from natural products and their derivatives [12]. Glucosinolates (GLSs) are important plant secondary metabolites present in the order of Brassicales [13]. GLSs are hydrolyzed by myrosinases forming various enzymatic hydrolysis products [14]. Isothiocyanates (ITCs), one type of hydrolysis products from GLSs, seem to be promising anticancer drugs, which have been proved to inhibit tumors by promoting autophagy, inducing epigenetic modification, and inhibiting glycolysis and fat metabolism in a growing number of studies [15,16,17]. Nowadays, some ITCs have entered clinical trials for the treatment of multiple cancer types, such as lung cancer, prostate cancer, and oral cancer [18,19,20].

In this review, we provide information on GLSs and its derived ITCs, and mainly discuss the mechanisms for different ITCs in inhibiting the carcinogenic properties of HCC.

## 2. Aetiology and Pathophysiology of HCC

### 2.1. Risk Factors for HCC

The incidence of liver cancer has continued to rise globally, posing a serious challenge to human health. As the major histological subtype of primary liver cancer, the occurrence of HCC is mostly relevant to chronic liver disease (more than 90% of cases), among which liver cirrhosis of any etiology is known to predispose toward HCC [21,22]. The proportion of liver cirrhosis developing into HHC reaches 1–6% every year, especially in patients with liver hepatitis or liver injury triggered by hepatitis virus B (HBV) and C (HCV) infection and unhealthy drinking, and HHC has also become one of the main causes of death in patients with liver cirrhosis [23,24,25,26]. The major risk factors for HCC include HBV and HCV infection, alcohol-related liver disease, type 2 diabetes, obesity-related non-alcoholic steatohepatitis and exposure to dietary, among which HBV and HCV infection are the most prominent risk factors, accounting for about 80% of HCC cases [8,27]. HBV is a DNA virus that can integrate into the host genome to induce insertion mutation, leading to oncogene activation [28]. Moreover, aflatoxin B1 exposure may have a synergistic effect with HBV to increase the risk of HCC [29,30]. However, timely hepatitis B birth dose vaccination has the potential to reduce HBV cases [31]. Unlike HBV, HCV is a RNA virus that does not integrate into the host genome and, therefore effective early detection is crucial for the treatment of HCV-infected patients [32]. Less common causes of HCC include age, sex, race and so on [33,34,35].

### 2.2. Pathophysiology

The occurrence and development of HCC are a complex multi-step process that usually occurs in the context of cirrhosis and is associated with a diversity of underlying liver diseases, including persistent inflammatory injury such as hepatocyte necrosis and regeneration, and fibrosis deposition [21,22,36]. The malignant transformation of liver cirrhosis into HCC follows a precise sequence of lesions: (i) from cirrhosis to low-grade dysplastic nodules, (ii) followed by high-grade dysplastic nodule, (iii) which subsequently transforms into early HCC and (iv) further results in progressed and eventually advanced HCC [37]. This carcinogenesis process involves multiple genetic aberrations in the molecular control of hepatocyte proliferation, differentiation and death, and the maintenance of genomic integrity [3,38]. The major pathways mutated in HCC include telomere maintenance, Wnt/*β*-catenin pathway, P53 cell cycle pathway, epigenetic modifiers, oxidative stress pathway, PI3K/AKT/MTOR and RAS/RAF/mitogen-activated protein kinase pathways [38]. The pathogenesis of HCC is associated with the cumulative activation and inactivation of oncogenes, tumor suppressor genes and other genes, as well as epigenetic alterations [8,39].

## 3. GLSs and Their Derived ITCs

**Figure 1:**
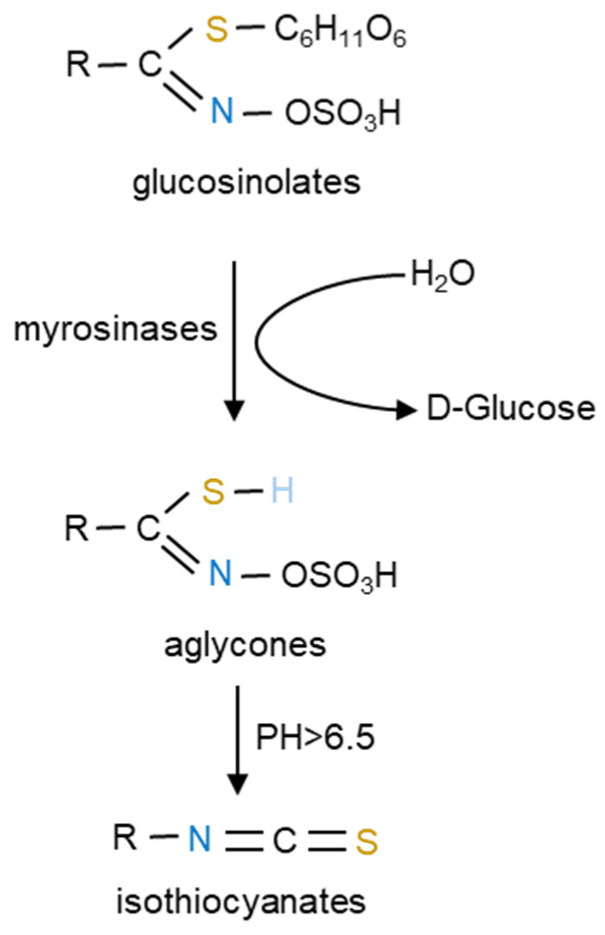
Glucosinolate hydrolysis into isothiocyanates by myrosinases. Glucosinolates (GLSs) are determined by a *β*-D-thioglucoside, N-hydroysulfates sulfur-linked to a sulfonate aldoxime and a variable side chain (R) derived from amino acids. GLSs generate aglycones under the action of myrosinases. As the structure of aglycones is unstable, ITCs are generated when the pH is greater than 6.5. Nitrogen, sulfur and hydrogen are indicated in blue, yellow and light blue, respectively.

Epidemiological studies have confirmed that intake of cruciferous vegetables in the diet helps reduce the risk of malignant tumors, attributed to the bioactive substances ITCs that are the hydrolysates of GLSs [40,41,42,43]. GLSs are a group of sulfur- and nitrogen-containing secondary metabolites, present primarily in the plant order Brassicales including Brassicaceae which contains several of daily vegetables, such as broccoli, cauliflower, cabbage, mustard, horseradish and white radish [44]. Chemically, GLSs share a common structure consisting of a *β*-D-thioglucoside, N-hydroxysulfates sulfur-linked to a sulfonate aldoxime and a variable side chain (R) derived from amino acids (Figure 1) [45]. To date, about 200 GLSs have been identified [46]. According to the structure of different amino acid precursors, GLSs are divided into arylaliphatic, aliphatic and indole GLSs [46,47].

During food preparation, chewing, and digesting, GLSs are broken down by *β*-thioglucosidase enzymes, known as myrosinases, into unstable aglycone moieties which rearrange to form bioactive compounds such as ITCs, nitriles, thiocyanates, and related compounds [48,49]. In intact plant tissues, GLSs and myrosinases are spatially separated, present in the vacuoles of so-called *S*-cells and in adjacent cells, respectively [50]. Upon plant tissue disruption, for instance, induced by cutting or chewing, GLSs come in contact with myrosinases to generate a hydrolysis in the presence of water [45,51]. The enzymatic hydrolysis of GLSs under the action of myrosinases into ITCs is shown in Figure 1.

ITCs are a family of compounds as the most intensively studied hydrolysates of GLSs at present, with -N=C=S considered as the most important active group [52,53]. There are in vitro and in vivo evidence that ITCs have multiple biological activities including plant defense and benefits to human health (antioxidant, antimicrobial and anticarcinogenic properties) [54,55,56,57,58]. ITCs have attracted much attention due to their potentially health-promoting activities associated with an anticarcinogenic activity in several organs, including lung, breast, colon, prostate, bladder and liver [59,60,61,62]. At present, the most extensively studied ITCs derived from GLS hydrolysis are allyl isothiocyanate (AITC), sulforaphane (SFN), benzyl isothiocyanate (BITC), phenethyl isothiocyanate (PEITC), 4-(methylthio) butyl isothiocyanate (4-MTB-ITC) and indole-3-carbinol (I3C, a breakdown product of indolic ITCs) [16,63,64,65,66,67]. Among these components, I3C and SFN have been most frequently examined for their anticancer effects [68,69]. In Table 1, we list the dietary sources, precursors and structures of ITCs covered by this review.

## 4. The Role of ITCs as Chemopreventive Agents on HCC

The preventive strategy to use naturally occurring or synthetic chemical agents to reverse, inhibit, or delay carcinogenesis when the hosts have been exposed to pathogenic factors is called chemoprevention [76]. Many epidemiological studies have provided strong evidence that a high intake of cruciferous vegetables and their constituents has a decreased risk of cancer [77,78]. As early as the 1970s, Wattenberg found that additions of ITCs to a diet could effectively inhibit chemical carcinogensis [79]. So far, a large number of studies have confirmed that ITCs play a significant chemopreventive role in various cancers, such as lung cancer, breast cancer, prostate cancer and liver cancer [80,81,82,83]. The doses of ITCs used for analysis of the chemopreventive potential in in vivo models varied considerably from 1 to 1100 mg/kg of body weight according to the specific type [84]. Nowadays, investigations of some individual ITCs reach the level of clinical trials for cancer prevention, such as SFN (ClinicalTrials.gov Identifiers: NCT03232138, NCT03517995, NCT01265953, NCT01228084 and NCT00946309) (Table 2) [85,86,87,88,89].

Considerable evidence suggests that ITCs could exert their cancer preventive effects by inhibiting the activation or enhancing the detoxification of the potential carcinogens or by acting on later stages of the carcinogenetic process, interfering with various distinct but interconnected signaling pathways involving modulating phase I and phase II enzymes, activation of nuclear factor erythroid 2-related factor 2 (Nrf2) signaling pathway and epigenetic regulation [17,78].

### 4.1. ITCs Inhibit the Activation and Enhance the Detoxification of Carcinogens by Modulation of Phase I and Phase II Enzymes

The metabolic activation of potential carcinogens primarily requires the catalysis by phase I and II biotransformation enzymes to cause DNA damage and cancer [84]. Phase I enzymes convert carcinogens through oxidation into active intermediates that easily bind to biological macromolecules such as DNA, RNA, and proteins [92]. Cytochrome P450 (CYP450) enzymes have proved to be the major phase I enzymes in the activation of potential pro-carcinogens such as aflatoxin B1, alpha-asarone, nitrosamines, polycyclic aromatic hydrocarbons [93,94,95,96,97]. Phase II enzymes, mainly including glutathione S-transferases, uridine 5′-diphospho-glucuronosyltransferases (UDP-glucuronosyltransferases), nicotinamide adenine dinucleotide phosphate (NADPH) quinone oxidoreductase 1 (NQO1), quinine reductases and glutamate cysteine ligase, have been implicated in detoxification of carcinogens, by promoting the conjugation of reactive intermediates with endogenous cofactors to produce water-soluble products and facilitating their excretion from the body through bile or urine [92,98].

In general, ITCs have been proposed to downregulate phase I enzymes to inhibit carcinogen activation and upregulate phase II enzymes to enhance detoxification and excretion of carcinogens, leading to the protection from carcinogenesis [98,99]. Relevant in vitro studies have directly proved the inhibitory effect of PEITC and SFN on CYP450 activities [100,101]. Dietary doses of SFN were demonstrated to depress the hepatic activity of *CYP1A2*, *CYP2B* and *CYP3A* for in vivo experiments in rats (Table 2) [90]. Besides, in rat hepatocytes, SFN alone has also proved to significantly enhance the *GSTA1* mRNA level in a dose-dependent manner, while co-treatment of SFN with *β*-naphthoflavone leads to a substantial increase in NQO1 activity and a marked decrease in *CYP1A1*, *CYP2B*, and *CYP3A4* expression, thus exerting its chemopreventive activity [102]. In addition, the chemopreventive effect of SFN on detoxication of the aflatoxin B1-8,9-epoxide in alpha mouse liver (AML) 12 cells has been reported to be associated with the upregulation of several GST isozyme genes [103]. Except SFN, addition of PEITC to rats at all dietary doses could markedly elevate the quinone reductase in liver tissues and stimulate the activity of hepatic GSTs (Table 2) [91]. Furthermore, Marca et al. treated primary rat hepatocytes with eight different ITCs (the aromatic benzyl, 4-hydroxybenzyl, phenethyl ITCs and the aliphatic allyl, napin, iberin, raphasatin ITCs, and SFN) and found that aromatic ITCs significantly increased the transcription of *CYP1A1* and *CYP1A2* mRNA and all these eight ITCs up-regulated most antioxidant/detoxifying enzymes, especially NADPH [104]. Collectively, all these findings support a chemopreventive effect for ITCs in liver.

Kelch-like ECH-associated protein-1 (Keap1)-Nrf2-antioxidant response element (ARE) signaling pathway represents one of the most important defense mechanisms against oxidative stress and exogenous toxic substances [105,106,107]. Nrf2 is an anti-oxidative stress regulator, which is sequestered in the cytoplasm by an inhibitor partner the cytoskeletal anchoring protein Keap1 through ubiquitination and degradation via the ubiquitin proteasome system under normal conditions [108,109]. Oxidative stress inducers dissociate this complex and cause dissociation of Nrf2 from Keap1 and subsequent translocation into the nucleus, triggering the induction of a verity of ARE driven detoxification enzymes and antioxidant factors, such as phase II enzymes [78]. Moreover, activating Nrf2 signaling plays a crucial role in prevention and treatment of various oxidative stress-related diseases including chemical carcinogenesis, metabolic and inflammatory diseases [110,111,112,113].

**Figure 2:**
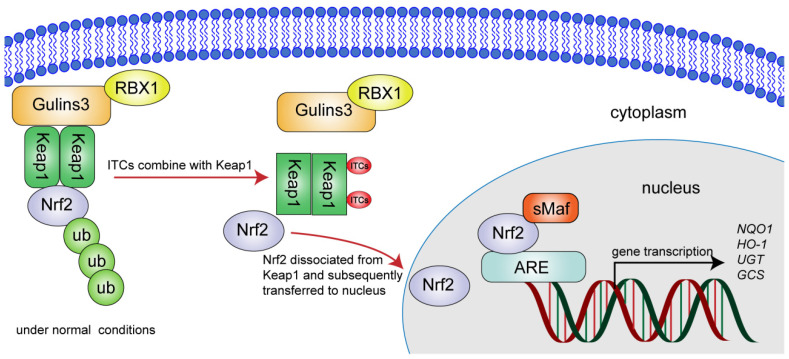
The regulatory effect of isothiocyanates on Keap1-Nrf2-ARE signaling pathway. Under normal conditions, Nrf2 is anchored into the cytoplasm by binding to Keap1, which facilitates the proteasomal degradation by ubiquitination. Under the action of the chemoprotective inducers ITCs, nascent Nrf2 translocates to the nucleus due to binding of ITCs to the sulfhydryl group of Keap1, and then binds to antioxidant response element (ARE) sequences in the nucleus, promoting the expression of genes and enzymes that regulate redox homeostasis. Red, purple, yellow, and light green ellipses represent ITCs, Nrf2, PBX1, and ub, while orange, dark green, blue, and red boxes represent Gulins3, Keap1, ARE, and sMaf, respectively. ub, ubiquitination; sMaf, small Maf proteins.

The ability to induce phase II and antioxidant enzymes via the Nrf2 signaling pathway has been also reported for ITCs as SFN, I3C, PEITC, AITC and BITC [114,115,116]. It was described that ITCs can bind to the sulfhydryl group of Keap1 to induce phase II enzymes, thereby preventing carcinogens and oxidants as showed in Figure 2 [117,118]. It is worth noting that the induction effect on Nrf2 and antioxidant enzyme *HO-1* in hepatoma cell varies with different ITCs. In HepG2, SFN not only strongly induced Nrf2 protein expression and ARE-mediated transcriptional activation, but also inhibited Keap1 to delay the degradation of Nrf2, thus activating the transcriptional expression of *HO-1*; AITC also induced the expression of Nrf2, ARE and *HO-1*, but had little effect on slowing down the degradation of Nrf2 protein; I3C could induce ARE-reporter gene expression and Nrf2 to some extent, but was not as potent as the formers [119]. Moreover, synergistic effects were observed in combination with I3C and SFN or PEITC in a human liver hepatoma cell line (HepG2-C8), leading to the induction of endogenous Nrf2, phase II genes (*GSTm2*, *UGT1A1* and *NQO1*) and antioxidant genes (*HO-1* and *SOD1*), which could ultimately enhance cancer chemopreventive activity [120].

### 4.2. Chemopreventive Activity of ITCs on HCC through Epigenetic Regulation

Epigenetic regulation, defined as heritable changes in gene expression that occur without alterations in DNA sequence, including DNA methylation, histone modification, and expression of microRNA (miRNA), plays a core role in the pathogenesis and chemoprevention of various cancers including HCC [121,122,123]. A large number of studies have found that ITCs are promising natural compounds in epigenetic targeted therapy [41,122,124].

#### 4.2.1. The Effect of ITCs on Post-Translational Histone Modification and DNA Methylation in HCC

The N-terminal of histone can undergo a variety of post-translational modifications, such as acetylation, methylation, phosphorylation and ubiquitination, which affect the structure and function of chromosome and finally play a role in the occurrence and development of cancer [125,126]. DNA methylation is an important aspect of epigenetics. A considerable number of experimental studies underline that hypermethylation of DNA causes changes in cell regulatory pathways, cell cycle and migration in tissues, resulting in HCC [127,128,129]. There is mounting evidence that the chemoprevention mechanism of ITCs depends on the changes of histone deacetylases (HDACs) and the inhibition of DNA methylation in various cancers [130]. Recently, Dos Santos et al. found that SFN played an epigenetic regulatory role in human hepatoma cells (HepG2) by inhibiting HDACs and might affect the activity of oncogenic transcription factor through methylation of its binding site motifs, offering insights into SFN chemopreventive molecular effects [131].

#### 4.2.2. The Ability of ITCs to Alter miRNA Expression in HCC

Besides the mentioned epigenetic regulatory mechanism to inhibit the activity of HDACs, the influence of ITCs on miRNA expression and modulation is also important. MiRNA is a class of small endogenous RNAs that regulate gene expression after transcription [132]. There is existing evidence to substantiate that multiple ITCs are capable to modulate miRNA expression in tumor cells, such as PEITC, SFN, and BITC [133,134,135]. Some studies suggested that miR-21 was upregulated in HCC [136,137,138]. It has been reported that I3C acted as a miR-21 regulator, leading to the suppression of miR-21 and repression of the tensin homologue protein (PTEN)/AKT pathway, a potential therapeutic target against metastasis, thus inhibiting tumorigenicity of HCC cells [139].

## 5. Anticancer Effects and Molecular Mechanisms of ITCs on HCC

Since the surprising discovery in 1977 of the anticancer properties of ITCs, accumulating evidence from encouraging in vitro and in vivo animal models has supported that ITCs could inhibit HCC by inhibiting cell proliferation, promoting apoptosis, inhibiting cell migration, inducing autophagy and so on, dysregulating diverse proteins and signaling pathways (Table 3).

### 5.1. Inhibition of the Proliferation of HCC Cells

Infinite proliferation is one of the main characteristics of tumor cells, and inhibition of tumor cell proliferation has always been one of the main anti-tumor mechanisms [161,162]. ITCs could inhibit the abnormal proliferation of HCC cells through a variety of mechanisms, such as promoting apoptosis, inducing cycle arrest and regulating related proteins [40,163,164]. BITC was suggested to suppress survivin expression and activate apoptosis, ultimately inhibiting the proliferation of HCC Bel7402 and BLE cells in a dose-time dependent manner [140]. Iberin, SFN and Alyssin were found to induce the accumulation of intracellular reactive oxygen species (ROS) and arrest cells in S and G2/M phase to block proliferation in HepG2 [165]. It was also demonstrated that AITC and its N-acetylcysteine conjugate (a major metabolite of AITC) suppressed the proliferation of SK-Hep-1 human hepatoma cells by inhibiting invasion, migration and MMP-2/-9 activity [152].

### 5.2. Arrest of HCC Cell Cycle

The cell cycle directly regulated by cyclins and cyclin-dependent protein kinases (CDKs) is a highly ordered set of events related to eukaryotic cell replication [166]. In general, cell cycle is divided into four stages: G1, S, G2 and M [167]. A series of reasons such as abnormal expression of cyclins or abnormal DNA replication would lead to the cycle disorder of tumor cells, which has been an important strategy to inhibit the growth of cancer cells [62,143,168]. The inhibition effect of ITCs on HCC cell cycle is obvious but varies according to the specific type and dose. It has been reported that SFN with different doses can block the cell cycle of human HCC HepG2 cell line through distinct periods of stagnation. The HepG2 cell population was increasingly arrested at the sub G0/G1 phase with SFN (33.8 μM) treatment in a time-dependent manner [143]. By contrast, SFN at 8 μM for 24 h treatments on the HepG2 induced G2/M cell cycle arrest and upregulated the expression of *CDKN1A*, *CDK1*, and *CCNB1* that controls the DNA damage checkpoint [131]. In addition, AITC was uncovered to block the cell cycle of HepG2 in G2/M by regulating cyclin B1 [141].

### 5.3. Inducing Apoptosis of HCC Cells

Apoptosis refers to a genetically determined process of spontaneous and orderly death of cells to maintain the stability of the internal environment under physiological or pathological conditions [169]. Apoptosis pathways could be divided into exogenous death receptor (DR) pathway, endogenous mitochondrial pathway, and endogenous endoplasmic reticulum (ER) pathway [170]. Meanwhile, granzyme B has been also implicated in the mediation of apoptosis process under certain conditions [171]. Several studies have reported that ITC-targeted apoptosis pathways play an important role in the treatment of cancer [172,173,174].

#### 5.3.1. Apoptosis Process Mediated by Mitochondria

When cells are suffering from apoptosis-stimulating factors or activated by death ligand, B-cell lymphoma-2 (Bcl-2) family proteins as Bak and Bax are activated, governing the membrane potential reduction and mitochondrial outer membrane permeabilization (MOMP), which further cause the release of cytochrome C and other apoptotic factors from mitochondria to cytoplasm, caspase apoptosis pathway activation and cell self-destruction [175]. Anticancer properties of ITCs promote activation of mitochondria-mediated apoptosis in various types of cancer cells, including HCC. Wu et al. treated PLC/PRF/5 cells with 5 μM PEITC and found that it could activate mitochondrial signal, release cytochrome C, reduce mitochondrial membrane potential, and then activate caspase-3/-9/-8 to cause apoptosis [145]. In addition, PEITC promoted the protein levels of tumor suppressor p53, which has been demonstrated to directly affect mitochondrial outer membrane permeability [145,176]. Wasabia japonica extract containing 5-(methylsulfinyl) pentyl ITC, 6-(methylsulfinyl) hexyl ITC and 7-(methylsulfinyl) Heptyl ITC could induce the accumulation of ROS and decrease the mitochondrial membrane potential, causing mitochondrial apoptotic pathway [148].

#### 5.3.2. Apoptotic Pathway Induced by Endoplasmic Reticulum Stress

Endoplasmic reticulum stress (ERS) is defined as the accumulation of unfolded or misfolded proteins in the ER under endogenous or exogenous disturbance factors, that activate a series of complex signaling pathways [177,178]. Excessive ERS triggers apoptotic signals and has been considered to be the pretty important cause of apoptosis [179,180,181]. Multiple relevant studies have confirmed that ITCs such as AITC, BITC, PEITC, and SFN could generate anticancer activity through ERS-mediated apoptosis [172,182,183,184]. As for this pathway, the increased expression of Bip/GRP78 and XBP-1 is a marker [185,186]. Zou et al. have found that SFN treatment with 20–40 μM for 48 h significantly inhibited the proliferation of HepG2 cells and upregulated the protein levels of Bip/GRP78, XBP-1, caspase-12, CHOP/GADD153, and Bid, proving that ERS is the most important mechanism of SFN-induced apoptosis of HepG2 cells [149].

#### 5.3.3. Apoptosis Process Mediated by Death Receptors

DRs belong to the tumor necrosis factor (TNF) receptor superfamily, including Fas, TNFR1, DR4, DR5 and DR3 [187]. When DR binds to the corresponding death ligands, it initiates a series of signal transduction and activates downstream caspase signal pathway, inducing apoptosis [188]. Currently, the best studied apoptotic DR signaling pathways include Fas/Fas ligand (FasL), tumor necrosis factor related apoptosis-induced ligand (TRAIL) and tumor necrosis factor receptor (TNFR) [189,190,191]. Yang et al. clarified that SFN could activate Fas signaling pathway and induce anoikis apoptosis in HepG2 and SMMC7721 cells by downregulating keratin 8 and keratin 18 (K8/18) [192]. TRAIL has become a promising new anticancer biotherapeutic. Relevant studies have found that I3C sensitizes HepG2 cells to TRAIL-induced apoptosis mainly through upregulation of caspase-3 activity, DR4 and DR5 expression, and down-regulation of Bcl-2 expression [193].

**Figure 3:**
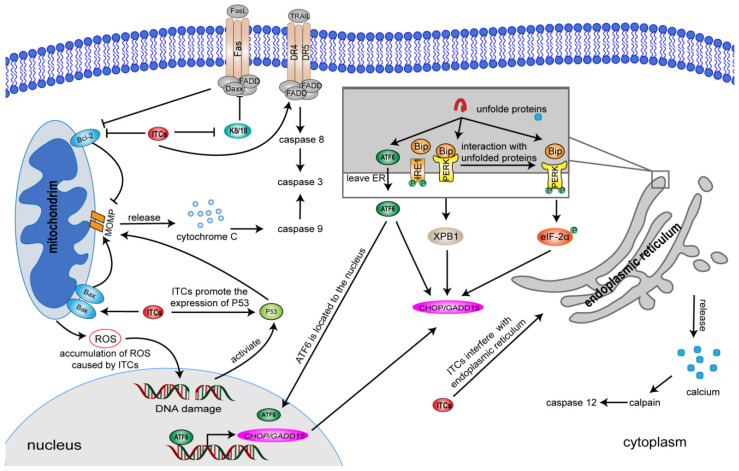
Regulatory pathways of apoptosis induced by isothiocyanates in hepatocellular carcinoma cells. Isothiocyanates (ITCs) mainly induce three apoptotic pathways in HCC. (i) ITCs trigger a series of mitochondria-related apoptotic responses, including decrease of mitochondrial membrane potential, increase of Bax expression and decrease of Bcl-2 expression, ultimately promoting the enhancement of mitochondrial outer membrane permeabilization (MOMP), which further causes the release of cytochrome C and promotes the production of ROS that causes DNA damage. (ii) ITCs induce endoplasmic reticulum stress (ESR)-related apoptosis pathway. As for this pathway, PERK and IRE1 are separated from molecular chaperones such as Bip/GRP78 due to the interaction between the unfolded/misfolded protein and molecular chaperones, and activated by autophosphorylation, promoting the production of elF-2*α* and expression of XBP1. Elf-2*α* and XBP1 further upregulate the levels of apoptosis signaling molecule CHOP/GADD153. Besides, ATF6 is also stimulated to transfer into the nucleus and promotes the transcription and expression of CHOP/GADD153. Moreover, a large amount of Ca^2+^ is released to enter the cytoplasm and activate calpain and caspase-12. (iii) ITCs induce apoptosis process mediated by TNF-related apoptosis-inducing ligand (TRAIL) and Fas/Fas ligand (FasL). ITCs inhibit keratin 8 and keratin 18 (K8/18), leading to the binding of FasL homotrimer (FADD, Daxx, and FAD-1) complex with Fas, which initiates Fas-FasL-mediated apoptosis of external death receptor pathway. In addition, ITCs induce TRAIL binding to DR4 and DR5, activating caspase-3 and mitochondria-dependent pathways to facilitate apoptosis. Red, blue, light green, dark green, pink, orange, gray, and reddish ellipses represent ITCs, Bcl-2/Bax, p53, ATF6, CHOP/GADD15, Bip, XPB1, and eIF-2α, respectively, while red hollow circle represents ROS.

The ITC-induced regulatory pathways of apoptosis in HCC described above are represented in Figure 3.

### 5.4. Inhibition of Tumor Cell Migration

Tumor invasion and metastasis as the primary causes of death for cancer patients correlate with the expression of matrix metalloproteinases (MMPs) and their inhibitors (TIMPs) [194]. MMPs, a kind of Zn^2+^- and Ca^2+^-dependent proteolytic enzymes, are responsible for extracellular matrix degradation and tissue remodeling, promoting the angiogenesis of tumor cells and the invasion and metastasis of adjacent tissues [195]. Currently, several studies have proved that ITCs downregulate the expression of MMP-2/-9 and upregulate the expression of TIMP1/2 in HCC in vitro, finally resulting in the inhibition of HCC progression [140,152,153,196]. It has been found that BITC treatment inhibited the MMP-2/-9 protein expression in a dose-dependent manner, whereas it increased TIMP-2 expression in SK-Hep-1 human hepatoma cells [155]. Further investigation revealed that the anti-metastatic activities of BITC might be achieved by the suppression of the phosphorylation activity of mitogen-activated protein kinases (MAPKs) [155]. Furthermore, BITC significantly inhibited the expression of MMP-2 in Huh 7 and Hep G2 and exerted the antitumor effect on HCC either in-vivo or in-vitro through suppressing HGF/pAKT/STAT3 axis [83]. Apart from BITC, AITC has been also reported exhibiting antimetastatic activity [154].

### 5.5. Inhibition of Tumor Cell Angiogenesis

Angiogenesis-related pathways play an important role in the HCC progression [197]. The VEGFs and their receptors (VEGFRs) are prime regulators in angiogenesis both physiologically and pathologically [198,199]. It was found that treating HCC with BITC significantly inhibited the release of angiogenesis marker VEGF either as in-vivo or as in-vitro, indicating that BITC could retard HCC progression by blocking cancer angiogenesis [83]. In addition, PEITC treatment remarkably suppressed the secretion of VEGF and the accumulation of hypoxia-inducible factor-1 (HIF-1*α*) in HepG2 during hypoxia through phosphotylinosital 3 kinase (PI3K) and MAPK signaling pathways [156]. The anti-angiogenesis and anti-tumor effects of SFN on HCC HepG2 cells through inhibition of STAT3/HIF-1*α*/VEGF signaling have been also demonstrated [157].

### 5.6. Decrease in Telomerase Activity of Tumor Cells

Telomerase is a specific reverse transcriptase that maintains telomeres on the ends of chromosomes [200]. Telomerase is frequently overexpressed in cancer cells and its activation is necessary for the continued development of some human cancers [201]. There is evidence of a close relationship between the inhibitory effect of SFN on HCC and telomerase activity, as Moon et al. found that the transcriptional and posttranslational regulation of telomerase reverse transcriptase (hTERT) was involved in SFN-induced suppression of telomerase activity in Hep3B cells via the ROS-dependent pathway [158]. Moreover, the DNA inhibitor MTBITC has also been implicated in the effective inhibition of telomerase activity both in vivo and in vitro. It has been found that, after MTBITC treatment, MAPK signaling pathway in Hep3B, HepG2 and Huh7 cells was activated, which increased the expression of *hTERT* mRNA and down-regulated telomerase activity, ultimately leading to apoptosis of HCC cells [159]. Furthermore, in an orthotopic human HCC xenograft model, Herz et al. demonstrated for the first time that MTBITC significantly reduced telomerase activity in vivo [160].

## 6. Sensitization to Chemotherapeutic Agents or Radiation Therapy by ITC Pre- or Co-Treatment

HCC is characterized by high drug resistance, easy metastasis, and high relapse rate after cure [8,202]. As broad-spectrum anticancer natural products, ITCs combined with other radiotherapy drugs have been clarified to greatly increase the anti-HCC efficacy [203,204,205]. A combination of moringin (a glycosyl-isothiocyanate, MOR) and avenanthramide (AVF-2f) has been proved an effective chemopreventive cocktail against HCC. This therapy inhibits Hep3B proliferation through exogenous and endogenous apoptosis, in which MOR triggers endogenous apoptosis pathway by the increase of ROS level and activation of caspase-2/-9, while AVF-2f induces exogenous pathway by the activation of caspase-8 expression [203]. Ren et al. found radiation increased the activity of NF-*κ*B in HCC cells, and combined treatment with the NF-*κ*B inhibitor PDTC induced HCC cell death. The subsequent combination of radiation and SFN with HCC cells achieved the same efficacy. It was further proved that SFN enhances the radiosensitivity of HCC by blocking the NF-*κ*B pathway both in vitro and in vivo [206]. Due to insufficient uptake and non-specific distribution, cisplatin has low chemical sensitivity and obvious side effects, which greatly limits its clinical application [207]. Recently, SFN has been reported to restore cisplatin chemosensitivity in HCC HepG2 cells by scavenging glutathione [205].

## 7. Conclusions and Perspectives

ITCs are hydrolysates derived from secondary metabolites GLSs of cruciferous vegetables. Considerable evidence supports the chemopreventive and anticancer activities of ITCs on HCC, making them promising candidates for novel anti-HCC drugs. The effectiveness of ITCs in the chemoprevention of HCC corelates with modulation of detoxifying enzymes and epigenetic regulation. ITCs exert anticancer activity in HCC by interfering with diverse proteins and signaling pathways implicated in cell cycle, apoptosis and metastasis as well as some other processes. In addition, the combination of ITCs and other chemotherapeutic agents in HCC could significantly enhance the therapeutic effect by upregulating the apoptotic pathway and detoxification, which indicates that ITCs are a good anticancer adjuvant drug. However, so far, no ITCs have entered clinical trial stage for the treatment of HCC, partially attributed to the deficency of sophisticated feasible treatment options containing the type and dose of ITCs. In addition, the exact molecular mechanisms of ITC action in HCC, especially the epigenetics, key molecular structures and action targets of ITCs, remain largely outside our realm of cognition. The combination of ITCs with radiotherapy, chemotherapy drugs and immunotherapy, as a valuable breakthrough point for HCC treatment, also lacks sufficient experimental support from in vivo and in vitro models.

## Figures and Tables

**Table 1:** Information on dietary sources of isothiocyanates and their glucosinolate precursors.

Isothiocyanates	Structures	Glucosinolate Precursors	Dietary Sources	References
Allyl isothiocyanate (AITC)	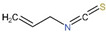	Sinigrin	Broccoli, brussels sprouts, and brassicanigra	[70]
Sulforaphane (SFN)	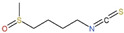	Glucoraphanin	Broccoli	[71]
Benzyl isothiocyanate (BITC)	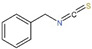	Glucotropaeolin	Watercress, cabbage, and broccoli	[72]
Phenethyl isothiocyanate (PEITC)	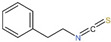	Gluconasturtiin	Broccoli, brussels sprouts, and watercress	[73]
4-(methylthio) butyl isothiocyanate (4-MTB-ITC)	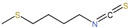	Glucoerrucin	Rocket salad	[74]
Indole-3-carbinol (I3C, a derivative of indolic ITCs)	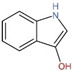	Glucobrassicin	Cabbage, broccoli, brussels sprouts, and cauliflower	[75]

**Table 2:** Chemopreventive activity of isothiocyanates on hepatocellular carcinoma and other cancers in in vivo models.

Isothiocyanates	Types of Cancers	ClinicalTrial.gov Identifiers ^a^	Doses	References
SFN	Lung cancer	NCT03232138	Oral-120 μM/day	[88]
SFN	Bladder cancer	NCT03517995	Oral-200 μM/day	[86]
SFN	Prostate cancer	NCT01265953	Oral-200 μM/day	[89]
SFN	Prostate cancer	NCT01228084	Oral-200 μM/day	[85]
SFN	Prostate cancer	NCT00946309	Oral-100 μM/day	[87]
SFN	Liver cancer	/	12 mg/kg	[90]
PEITC	Live cancer	/	0.6–6.0 μM/g	[91]

^a^ represents no ClinicalTtrial.gov Identifier.

**Table 3:** Anticancer effects of isothiocyanates on hepatocellular carcinoma and their molecular targets in in vitro and in vivo models.

Isothiocyanates	Inhibitory Approaches	Molecular Targets ^a^	Experimental Models	Concentrations	References
BITC	Inhibit cell proliferation	Survivin↓	Bel7402 and HLE	20 μM	[140]
AITC	Inhibit cell proliferation	Survivin↓	HepG2	2 and 5 μM	[141]
MTBITC	Arrest cell cycle	G2/M phase arrest	HepG2	25 μM	[142]
AITC	Arrest cell cycle	G2/M phase arrest Cyclin B1↓, *p53*↑, and *p21*↑	HepG2	2 and 5 μM	[141]
SFN	Arrest cell cycle	Sub G0/G1 phase arrest	HepG2	33.8 μM	[143]
SFN	Arrest cell cycle	G2/M phase arrest	HepG2	8 μM	[131]
SFN	Arrest cell cycle	S; G2/M phase arrest	HepG2	40 μM	[144]
Iberin	Arrest cell cycle	S; G2/M phase arrest	HepG2	40 μM	[144]
Alyssin	Arrest cell cycle	S; G2/M phase arrest	HepG2	40 μM	[144]
PEITC	Induce cell apoptosis	caspase-9/-3/-8↑, Bax↑, *p53*↑, Bcl-2↓, BclXL↓, and cytochrome C↓	PLC/PRF/5 cells	5 μM	[145]
*β*-PEITC	Induce cell apoptosis	caspase-9/-3↑, Bax↑, mitochondrial membrane potential↓, cytochrome C↓	HepG2	20μM	[146]
SFN	Induce cell apoptosis	caspase-3↑, Bcl-2, BclXL↓, and Bax↑	HepG2	20 μM	[147]
MTBITC	Induce cell apoptosis	Caspase-3/-7↑	HepG2	25 μM	[142]
Wasabia japonica extract contained 5-(methylsulfinyl) pentyl ITC, 6-(methylsulfinyl) hexyl ITC, and 7-(methylsulfinyl) heptyl ITC)	Induce cell apoptosis	ROS↑ and *p73*↑	Hep3B	0.25 to 1 mg/mL	[148]
Wasabia japonica extract contained 5-(methylsulfinyl) pentyl ITC, 6-(methylsulfinyl) hexyl ITC, and 7-(methylsulfinyl) heptyl ITC)	Induce cell apoptosis	ROS↑ and *p73*↑	Xenograft tumors	5 mg/kg	[148]
BITC	Induce cell apoptosis	caspase-3↑ and PARP-1↑	Bel7402	20 μM	[140]
AITC	Induce cell apoptosis	caspase-3/-8↑ and Bcl-2↓	HepG2	2 and 5 μM	[141]
SFN	Induce cell apoptosis	Bip/RP78↓, XBP-1↓, caspase-12↓, CHOP/GADD153↓, and Bid↓	HepG2	40 μM	[149]
SFN	Induce cell apoptosis	caspases-3/7/-9↑ caspases-8↓	HepG2	33.8 μM	[143]
MTBITC	Induce cell apoptosis	ROS↑	HepG2	10, 20, and 40 μM	[150]
Erysolin	Induce cell apoptosis	ROS↑	HepG2	10, 20, and 40 μM	[150]
PEITC	Induce cell apoptosis	ROS↑	HepG2	10, 20, and 40 μM	[150]
SFN	Induce cell apoptosis	ROS↑	HepG2	10, 20, and 40 μM	[150]
Sulforaphene	Induce cell apoptosis	ROS↑	HepG2	10, 20, and 40 μM	[150]
I3C	*Induce cell apoptosis*	*p53*↑, PARP↑, and caspase-3/-7↑	SNU449	300 μM	[151]
AITC	Inhibit cell migration	MMP-2/-9↓	SK-Hep-1	5 μM	[152]
AITC	Inhibit cell migration	MMP-2/-9↓, integrin*α*5*β*1↓	HepG2	2, and 5 μM	[141]
PEITC	Inhibit cell migration	MMP-2/-9↓ and TIMP1/2↑	SK-Hep-1	5 μM	[153]
AITC	Inhibit cell migration	COL8A1↓, COL4A3↓, and MMP-2/-9↓	SK-Hep-1	10 μM	[154]
I3C	Inhibit cell migration	miR-21↓ and PTEN↑	SK-Hep-1 and SUN449	200 μM	[139]
BITC	Inhibit cell migration	MMP-2/-9↓ and CXCR4↓	Bel7402	20 μM	[140]
BITC	Inhibit cell migration	MMP-2/-9↓	SK-Hep-1	0.1, 1, and 5μM	[155]
AITC	Inhibit cell migration	MMP-2/-9↓ and AKT/NF-*κ*B pathway	HepG2	2, and 5 μM	[141]
PEITC	Inhibit cell angiogenesis	HIF-1*α*↓ and VEGF↓	HepG2	10 μM	[156]
MTBITC	Inhibit cell angiogenesis	microtubule depolymerization	HepG2	10, 20, and 40 μM	[150]
Erysolin	Inhibit cell angiogenesis	microtubule depolymerization	HepG2	10, 20, and 40 μM	[150]
PEITC	Inhibit cell angiogenesis	microtubule depolymerization	HepG2	10, 20, and 40 μM	[150]
SFN	Inhibit cell angiogenesis	microtubule depolymerization	HepG2	10, 20, and 40 μM	[150]
Sulforaphene	Inhibit cell angiogenesis	microtubule depolymerization	HepG2	10, 20, and 40 μM	[150]
I3C	Inhibit cell angiogenesis	*p53*↑, PARP↑, and caspase-3/-7↑	SNU449	300 μM	[151]
SFN	Inhibit cell angiogenesis	STAT3↓, HIF-1*α*↓, and VEGF↓	HepG2	20 μM	[157]
SFN	Decrease telomerase activity	hTERT↑ and ROS↑	Hep3B	20 μM	[158]
MTBITC	Decrease telomerase activity	MAPK and hTERT↑	HepG2, Hep3B, and Huh7	25 μM	[159]
MTBITC	Decrease telomerase activity	telomerase activity↓	Xenograft tumors	50 mg/kg	[160]

^a^ ↑ and ↓ represent enhanced and suppressed gene expression and/or protein levels, respectively.
